# Modulating the Gut Microbiota via Rectal Ozone Insufflation in Gynecological Cancer Patients with Radiotherapy/Chemotherapy-Induced Pelvic Toxicity: A Proposed Clinical Study Protocol

**DOI:** 10.3390/jcm14228015

**Published:** 2025-11-12

**Authors:** Bernardino Clavo, Elizabeth Córdoba-Lanús, Gregorio Martínez-Sánchez, Mario Federico, Ángeles Cánovas-Molina, José E. Piñero, Ana M. Vargas-Prado, Avinash Ramchandani, Marta Zajac, Ivone Ribeiro, Minerva Navarro, Ignacio J. Jorge, Jesús M. González-Martín, Ruth Martín-Alfaro, María Fernández-Tagarro, Juan A. Díaz-Garrido, Jacob Lorenzo-Morales, Francisco Rodríguez-Esparragón

**Affiliations:** 1 Research Unit, Hospital Universitario de Gran Canaria Dr. Negrín, 35019 Las Palmas de Gran Canaria, Spain; mariofedericos@yahoo.it (M.F.); canovaspi@hotmail.com (Á.C.-M.); josu.estadistica@gmail.com (J.M.G.-M.); juanantdiaz@hotmail.com (J.A.D.-G.); afrodesp@gmail.com (F.R.-E.); 2 Chronic Pain Unit, Dr. Negrín University Hospital, 35019 Las Palmas de Gran Canaria, Spain; minervanavarrorivero@gmail.com (M.N.); ijja36@gmail.com (I.J.J.); 3 Radiation Oncology Department, Hospital Universitario Dr. Negrín, 35019 Las Palmas de Gran Canaria, Spain; marta.zajac23@gmail.com (M.Z.); ivone.ribeiro.gmr@gmail.com (I.R.); 4 Fundación Canaria Instituto de Investigación Sanitaria de Canarias (FIISC), 35019 Las Palmas de Gran Canaria, Spain; gregorcuba@yahoo.it; 5 University Institute for Research in Biomedicine and Health (UIBS), Molecular and Translational Pharmacology Group, University of Las Palmas de Gran Canaria, 35016 Las Palmas de Gran Canaria, Spain; 6 Instituto Universitario de Enfermedades Tropicales y Salud Pública de Canarias, Universidad de La Laguna, 38296 La Laguna, Spain; acordoba@ull.edu.es (E.C.-L.); jpinero@ull.edu.es (J.E.P.); 7 CIBER de Enfermedades Infecciosas (CIBERINFEC), Instituto de Salud Carlos III, 28029 Madrid, Spain; 8 Spanish Group of Clinical Research in Radiation Oncology (GICOR), 28290 Madrid, Spain; 9 Independent Researcher, 60126 Ancona, Italy; 10 Medical Oncology Department, Hospital Universitario de Gran Canaria Dr. Negrín, 35019 Las Palmas de Gran Canaria, Spain; avarpra@gobiernodecanarias.org; 11 Medical Oncology Department, Complejo Hospitalario Universitario Insular Materno-Infantil de Gran Canaria, 35016 Las Palmas de Gran Canaria, Spain; avirv87@hotmail.com; 12 Faculty of Health Sciences, Universidad Fernando Pessoa Canarias, 35450 Las Palmas de Gran Canaria, Spain; 13 Clinical Analysis Department, Hospital Universitario Dr. Negrín, 35019 Las Palmas de Gran Canaria, Spain; rmaralf@gobiernodecanarias.org (R.M.-A.); mfertag@gobiernodecanarias.org (M.F.-T.); 14 Department of Psychiatry, Hospital Universitario Dr. Negrín, 35019 Las Palmas de Gran Canaria, Spain; 15 Faculty of Psychology, Universidad Fernando Pessoa Canarias, 35450 Las Palmas de Gran Canaria, Spain

**Keywords:** ozone therapy, gut microbiota, gynecological cancer, radiotherapy toxicity, chemotherapy toxicity, dysbiosis, supportive care

## Abstract

Background: Chronic pelvic toxicity induced by radiotherapy and/or chemotherapy (R/CIPT) is a debilitating sequela in gynecological cancer survivors, often refractory to conventional treatments and potentially linked to gut microbiota dysbiosis. Ozone therapy (OT), particularly rectal insufflation, demonstrates anti-inflammatory and redox-modulating effects through hormetic mechanisms (Nrf2 activation/NF-κB inhibition). We hypothesize that its clinical benefit is mediated, in part, by restoring gut microbial homeostasis. Objective: This manuscript proposes a clinical study to evaluate the impact of rectal OT on the gut microbiota of patients with gynecological cancers and chronic R/CIPT. Proposed Methods: A prospective, observational study of 38 patients is outlined: 19 with CTCAE v5.0 Grade ≥2 chronic R/CIPT receiving compassionate rectal OT (~40 sessions over 4 months), and 19 matched controls without toxicity. Stool samples for 16S rRNA sequencing will be collected from the OT group pre- and post-intervention and once from controls. Primary endpoints are changes in microbiota composition/diversity and pelvic toxicity scores (CTCAE v5.0, EORTC QLQ-CX24). Secondary endpoints include quality of life (EORTC QLQ-C30, EQ-5D-5L), anxiety/depression (HADS), and serum inflammatory/oxidative stress biomarker analysis. Anticipated Results and Conclusion: This will be the first study to prospectively investigate whether rectal OT’s effect correlates with a beneficial shift in the gut microbiota, specifically an increase in commensals (e.g., short-chain fatty acids producers) and a decrease in pathobionts. If successful, OT could be assessed as a novel, microbiota-targeting intervention for R/CIPT. The findings from this pilot study will provide the necessary groundwork for a future randomized controlled trial to definitively establish causality and efficacy.

## 1. Introduction

Gynecological cancers, especially malignancies of the endometrium, uterine cervix, vagina, and vulva, represent a significant health burden worldwide, with radiotherapy (RT) and chemotherapy (QT) forming the cornerstone of treatment strategies [1,2]. Despite their effectiveness in improving survival, these therapies are frequently associated with acute and chronic toxicities, particularly when the pelvic region is irradiated. Among these, genitourinary (such as chronic mucosal ulcers, gynecological pain, cystitis, and vesical bleeding) and gastrointestinal (GI) side effects (such as diarrhea, pelvic pain, enteritis, proctitis, and rectal bleeding) markedly impair patients’ quality of life. Many times, these symptoms are refractory to conventional treatments [3,4].

Emerging evidence suggests that the intestinal microbiota plays a central role in mediating host responses to oncological treatments [5,6]. Pelvic RT and cytotoxic QT induce profound dysbiosis, characterized by reduced microbial diversity and shifts in protective taxa. These alterations are increasingly recognized as contributors not only to local GI toxicity but also to systemic inflammation and variable therapeutic efficacy. Consequently, interventions capable of preserving or restoring a balanced gut microbiota during oncological treatment are of growing clinical interest [7,8].

Ozone therapy (OT), a medical approach based on controlled administration of an oxygen–ozone mixture, has gained attention as a supportive treatment in oncology [9]. Its pleiotropic effects include modulation of redox signaling, enhancement of tissue oxygenation, anti-inflammatory activity, and regulation of immune responses. Clinical studies have reported that OT can reduce fatigue, improve quality of life, and mitigate adverse effects associated with RT and QT [10,11]. More recently, preclinical studies suggest that ozone administration, particularly through rectal insufflation, may directly or indirectly influence the gut microbial ecosystem [12,13]. This observation is consistent with the therapy’s known capacity to modify mucosal oxidative balance, microcirculation, and local immune milieu.

To date, however, the relationship between OT, gut microbiota composition, and the mitigation of RT/QT-induced toxicity in gynecological patients remains largely unexplored. Understanding this interplay is crucial, as it may open new avenues for reducing treatment-related morbidity and optimizing patient outcomes. This manuscript aims to (i) review current knowledge on the impact of RT and QT on gut microbiota in gynecological cancers, (ii) explore mechanistic insights into how OT may modulate microbial and host responses, and (iii) propose a clinical framework to investigate OT as an adjuvant strategy for managing therapy-induced toxicity. By addressing these objectives, we intend to provide a scientific rationale for integrating microbiome analysis into supportive oncology protocols and highlight the potential of OT as a novel adjunctive tool.

## 2. Radiotherapy and Chemotherapy Effects on Gut Microbiota

### Evidence of Dysbiosis in Patients Undergoing Pelvic RT/QT

The human gut microbiota, a complex ecosystem of trillions of microorganisms, plays a fundamental role in maintaining host health. It is involved in nutrient metabolism, protection against pathogens, and the regulation of immune function. In oncology, RT and QT are cornerstone treatments, but they are notoriously damaging to healthy, rapidly dividing cells—including those lining the GI tract. The detrimental effects of these treatments on the gut microbiota, a state known as “dysbiosis”, are now recognized as a major contributor to side effects and a potential modulator of treatment efficacy [3,4]. This discussion will focus on the evidence linking pelvic RT and systemic QT to significant alterations in the gut microbiota, the mechanisms behind this dysbiosis, and its clinical consequences.

(a) *Defining the Insult: How RT and QT Disrupt the Gut Ecosystem.*

The mechanisms by which these therapies damage microbiota are multifaceted:

– Direct Cytotoxicity: Both RT and QT are designed to kill rapidly dividing cells. While their target is cancer, they also attack the rapidly renewing epithelial cells of the intestinal lining [14,15]. This damage compromises the integrity of the mucosal barrier, creating an inflamed environment that is hostile to beneficial, obligate anaerobic bacteria (e.g., *Faecalibacterium*, *Roseburia*) and favorable for pathobionts (potentially pathogenic bacteria) [16].
– Indirect Effects via the Host: The destruction of the intestinal epithelium leads to a cascade of events: Mucus Layer Thinning: Goblet cells are damaged, reducing mucin production. This depletes the primary habitat and food source for many commensal bacteria. Inflammation: Cell death triggers a potent inflammatory response, releasing antimicrobial peptides and reactive oxygen species that further reshape the microbial community. Immune Dysregulation: The gut microbiota is in constant cross-talk with the immune system. Therapy-induced damage to immune cells in the gut-associated lymphoid tissue (GALT) disrupts this delicate balance [15,17].

(b) *Evidence of Dysbiosis in Patients Undergoing Pelvic RT.*

Pelvic RT, used for cancers of the prostate, cervix, uterus, rectum, and anus, directly irradiates the lower GI tract, making it a prime model for studying microbiota disruption. The evidence for dysbiosis is robust and characterized by several consistent patterns:

– Loss of Microbial Diversity: A hallmark of a healthy gut is high species richness (alpha-diversity). Multiple studies have demonstrated a significant and rapid decrease in alpha-diversity in patients undergoing pelvic RT. This loss is considered a sign of ecosystem instability and is strongly correlated with the severity of GI symptoms, particularly diarrhea [18,19].
– Shifts in Key Bacterial Taxa. Depletion of Beneficial Genera: There is a consistent and dramatic reduction in beneficial, short-chain fatty acid (SCFA)-producing bacteria. Butyrate producers like *Faecalibacterium prausnitzii* and *Roseburia* spp. are particularly vulnerable. Their loss is directly linked to mucosal barrier breakdown and inflammation [18,20].
– Expansion of Pathobionts: Concurrently, there is an increase in opportunistic pathogens and pro-inflammatory bacteria. Commonly reported blooms include *Enterobacteriaceae* (e.g., *Escherichia*, *Klebsiella*) and an increased susceptibility to *Clostridium difficile* infection [21,22].
– Dose–Response Relationship: The degree of dysbiosis is often correlated with the radiation dose delivered to the rectum and bowel. Higher doses are associated with more profound microbial shifts and worse clinical toxicity [20].
– Persistence of Dysbiosis: Critically, these changes are not always transient. Studies following patients for months after RT have found that microbial diversity and the abundance of key beneficial species often fail to return to pre-treatment levels, suggesting long-term alteration of the gut ecosystem [18].

(c) *Evidence of Dysbiosis in Patients Undergoing Systemic QT.*

While pelvic RT causes localized damage, systemic chemotherapy affects the entire GI tract. The evidence for QT-induced dysbiosis is equally compelling, though the specific changes can vary based on the chemotherapeutic agent.

Agent-Specific Effects:

– 5-Fluorouracil (5-FU): Strongly associated with mucositis and a decrease in microbial diversity, particularly reducing SCFA producers [23].
– Irinotecan: Causes severe diarrhea and is linked to a bloom of *Enterobacteriaceae* and a reduction in beneficial *Firmicutes* [24].
– Cyclophosphamide: Interestingly, some chemotherapies like cyclophosphamide rely on a specific microbiota to stimulate anti-tumor immune responses. It can cause a translocation of specific Gram-positive bacteria to secondary lymphoid organs, priming pathogenic T-helper 17 (Th17) cells [25].

Common Themes in QT-Induced Dysbiosis:

– Reduced Diversity: Similar to RT, a drop in alpha-diversity is a common finding [23].
– Impact on Treatment Outcomes: The microbiota can influence QT efficacy and metabolism. For instance, some gut bacteria can inactivate chemotherapeutic drugs, while others can convert prodrugs into their active forms [26].

(d) *Clinical Consequences of Therapy-Induced Dysbiosis.*

The dysbiosis described above is not merely an observational finding; it has direct and serious clinical implications:

– RT/CT-Induced Diarrhea and Mucositis: The loss of SCFA producers and the expansion of pro-inflammatory bacteria directly contribute to these debilitating side effects [24].
– Infection Risk: The loss of “colonization resistance” allows for the overgrowth of opportunistic pathogens like *C. difficile* [27].
– Systemic Inflammation: A leaky gut and an abundance of LPS from Gram-negative bacteria can lead to bacteremia and systemic inflammation.

The evidence is clear: both pelvic radiotherapy and systemic chemotherapy induce profound and specific dysbiosis in the gut microbiota. This dysbiosis is a key driver of the acute and chronic toxicities that diminish patients’ quality of life. This growing understanding has opened exciting new avenues for supportive care in oncology, including:

– Microbiota as a Biomarker: Pre-treatment microbial signatures may predict a patient’s risk of developing severe toxicity [28].
– Microbiome-Targeted Therapies: Interventions like probiotics, prebiotics, and fecal microbiota transplantation (FMT) are being actively investigated to prevent or reverse therapy-induced dysbiosis [28].

## 3. Biological Mechanisms of Ozone Therapy

OT exerts its biological effects primarily through the controlled generation of reactive oxygen species (ROS) and lipid peroxides (LOPs) upon contact with biological fluids. These molecules act as secondary messengers, triggering redox-sensitive signaling pathways such as Nrf2/Keap1/ARE, which upregulate antioxidant defenses and restore redox homeostasis. The hormetic response to low-dose ozone is characterized by a transient, moderate oxidative stress that stimulates cellular adaptation without causing cytotoxicity [29,30,31,32,33].

OT enhances microcirculation and tissue oxygenation by improving rheological properties of blood, increasing erythrocyte flexibility, and promoting oxygen release to hypoxic tissues. Clinical studies have demonstrated increased tissue oxygenation following systemic ozone administration, particularly in ischemic conditions [34,35].

The anti-inflammatory and immunomodulatory properties of ozone are mediated by downregulation of pro-inflammatory cytokines (e.g., TNF-α, IL-1β) and upregulation of anti-inflammatory mediators. Ozone modulates immune cell function, including regulatory T lymphocytes, and shifts cytokine profiles toward an anti-inflammatory state. This is achieved through redox signaling and direct effects on immune cell populations [10,36,37].

Emerging preclinical evidence suggests that ozone therapy, especially via rectal insufflation or ozonated water, can directly and indirectly impact the gut microbiota. Ozone administration has been shown to increase the abundance of beneficial bacteria (e.g., Lactobacillus, Bifidobacterium), restore microbial diversity, and modulate microbial metabolites such as SCFAs and tryptophan derivatives. These changes contribute to improved intestinal barrier function and reduced inflammation in models of atherosclerosis and sepsis [12,13,38].

Overall, OT acts as a redox bioregulator, with pleiotropic effects on cellular signaling, tissue oxygenation, inflammation, immune modulation, and the gut microbial ecosystem [39].

The proposed rectal ozone therapy protocol utilizes concentrations and volumes consistent with established clinical practice, typically ranging from 10 to 40 μg/mL and 180 to 300 mL per session, with gradual titration based on patient tolerance. These parameters have demonstrated safety and efficacy in oncological patients, including those with refractory pelvic toxicity and QT-induced neuropathy, with no serious adverse events reported and only transient meteorism as a minor side effect [40,41,42,43,44]. The impact of ozone on gut microbiota in this context remains unexplored and is a novel focus of the present study.

## 4. Evidence Linking Ozone Therapy to Microbiota Modulation

A growing, yet still nascent, body of research suggests that one of the mechanisms of action of Medical OT may be through the modulation of the gut microbiota. The proposed hypothesis is grounded in ozone’s fundamental principle of hormesis: a low, precise concentration induces a transient, controlled oxidative stress that triggers a beneficial adaptive response in tissues [29,33]. This response is characterized by the activation of the Nrf2 pathway—a master regulator of antioxidant cellular defense—and the concurrent inhibition of the pro-inflammatory NF-ĸB pathway [10,36,37].

When applied via rectal insufflation, this hormetic effect is postulated to rebalance the gut ecosystem through several interconnected mechanisms. The controlled oxidative stress may exert a selective antimicrobial effect, inhibiting opportunistic, pro-inflammatory pathobionts that thrive in the dysbiotic environment created by RT/CT [10,29]. Simultaneously, by attenuating local inflammation and oxidative stress, OT helps restore the mucosal microenvironment, making it more favorable for the recovery of beneficial commensal bacteria, particularly SCFA producers [12,13]. Furthermore, the trophic and reparative effects of ozone on the damaged intestinal epithelium could enhance barrier function, reducing bacterial translocation and subsequent systemic inflammation. Ultimately, this “weeding and seeding” of the gut lumen favors the growth of a healthier, more resilient microbiota [38,45].

### 4.1. Preclinical Data: Insights from Animal Studies

Preclinical evidence linking OT to modulation of the gut microbiota is robustly supported by controlled animal studies using rectal ozone insufflation. In a murine model of atherosclerosis, rectal OT led to significant shifts in the gut microbiome, notably increasing the abundance of beneficial genera such as *Lactobacillus* and *Bifidobacterium*, while reducing pro-inflammatory taxa. These microbial changes were accompanied by increased levels of SCFAs (propionic and butyric acid) and decreased levels of harmful metabolites Trimethylamine (TMA) and Trimethylamine N-oxide (TMAO), suggesting a shift toward an anti-inflammatory gut environment [12].

Further evidence indicates that rectal ozone insufflation reverses sleep deprivation-induced dysbiosis, normalizing the Firmicutes/Bacteroidetes ratio and the abundance of Bacteroides, which is associated with reduced inflammation and improved cognitive function. This effect is linked to the restoration of the intestinal barrier and a reduction in pro-inflammatory cytokines in the colon and brain [46]. Additional animal studies using ozone water enemas in mice transplanted with fecal microbiota from COVID-19 patients demonstrated that OT improved gut dysbiosis, enhanced intestinal barrier function, and reduced systemic inflammation. These effects were mechanistically linked to activation of the SIRT1-Nrf2/HO-1 pathway, providing direct experimental support for the immunoregulatory and antioxidant impact of ozone on the gut ecosystem [13].

**Figure 1:**
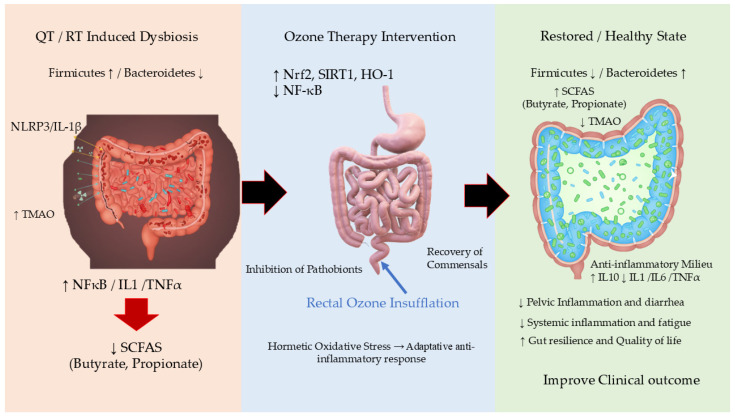
Proposed Mechanisms of Rectal Ozone Insufflation in Modulating the Gut Microbiome and Restoring Intestinal Homeostasis. This schematic illustrates the hypothesized pathway through which medical ozone, administered via rectal insufflation, exerts its effects. The core principle is oxidative hormesis, where controlled, low-dose ozone triggers a beneficial adaptive response. The process begins with (1) the local application of ozone in the gut lumen, which induces a mild, transient oxidative stress. This primary event initiates two key parallel mechanisms: (2) the Direct Modulation of the Gut Microbiota, and (3) the Activation of Host Cellular Pathways (Nrf2/SIRT1). These mechanisms converge to (4) Improve Gut Barrier Integrity and (5) Reduce Local and Systemic Inflammation, ultimately leading to the restoration of a healthy gut ecosystem and amelioration of disease symptoms. HO-1: Heme Oxygenase-1; IL-1β: Interleukin-1 beta; IL-6: Interleukin-6; IL-10: Interleukin-10; NF-κB: Nuclear Factor Kappa-Light-Chain-Enhancer of Activated B cells; NLRP3: NLR Family Pyrin Domain Containing 3; Nrf2: Nuclear Factor Erythroid 2–Related Factor 2. QT, Chemotherapy; RT, Radiotherapy; SCFAs: Short-Chain Fatty Acids; SIRT1: Sirtuin 1. TMAO: Trimethylamine N-Oxide; ↑, increase; ↓, Decrease.

Collectively, these studies demonstrate that OT, particularly via the rectal route, induces favorable changes in the diversity and composition of the gut microbiota, restores barrier function, and reduces inflammation, with effects that depend on the pathological context and the initial microbial profile (Figure 1) [12,13,46].

### 4.2. Emerging Clinical Observations and the Current Knowledge Gap

Despite the compelling preclinical rationale and the long-standing clinical use of rectal ozone (dating back nearly 90 years), direct evidence of its impact on the human gut microbiota remains a significant and original area of inquiry. In the oncology context, it is recognized that the gut microbiota influences immune response, inflammation, and the efficacy and toxicity of antineoplastic treatments [47,48]. However, clinical studies directly evaluating the effect of OT on the microbiota in cancer patients are scarce and, to date, no controlled trials demonstrating specific microbial changes following ozone administration have been published.

In other medical areas, such as rheumatoid arthritis and inflammatory diseases, clinical reports and reviews suggest that OT can induce an immunomodulatory and antioxidant response, which might be mediated, in part, by an interaction with the microbiota [49,50]. Nevertheless, robust data confirming microbiota changes in humans after OT are not yet available. This critical gap in knowledge underscores the novelty and relevance of the present project, which aims to be the first to prospectively investigate whether the documented clinical benefits of rectal ozone in patients with chronic pelvic toxicity are indeed mediated through a beneficial modulation of the gut microbiota.

## 5. Proposed Clinical Study/Protocol

Study Population. Primary Cohort (Group R/CIPT, Radiotherapy/Chemotherapy-Induced Pelvic Toxicity): Adult women (≥18 years) with gynecological malignancies (cervical, endometrial, vulvar, vaginal) who have received pelvic RT with/without CT and present with chronic pelvic toxicity (≥3 months duration) rated as Grade ≥2 according to the Common Terminology Criteria for Adverse Events (CTCAE) v5.0 from the U.S. National Cancer Institute. In our institution, these patients are usually referred for compassionate OT due to symptoms refractory to conventional treatment. Control Cohort: Gynecological cancer patients matched by age (±5 years) and primary tumor location, treated with R/CIPT but without symptoms of pelvic toxicity.

Intervention: Rectal Ozone Insufflation +/− direct local application to the affected mucosa (rectal, vesical, and/or vaginal), based on the standard treatment protocol of the Chronic Pain Unit for compassionate use of OT. Proposed Regimen: It usually consists of 40 sessions administered over 4 months, following the unit’s standard operating procedures. The ozone concentration (µgO_3_/mLO_2_) will start at 10 µg/mL and increase by 5 µg/mL every 2 sessions until it reaches 30 µg/mL by the 9th session, after which it will be maintained until week 16. The volume will start at 180 mL and will be progressively increased to 300 mL if tolerated. The total ozone dose will range from 1800 µg to 9000 µg. To address standardization, the rectal ozone insufflation procedure will use a sterile, flexible catheter (14–16 Fr, 20–25 cm), inserted 7.5–12 cm into the rectum after lubrication. The patient will be positioned on their side, and the oxygen-ozone mixture will be administered over 5 min. All procedures will follow the unit’s standard operating protocols and be performed by trained staff. This approach is supported by published clinical protocols and safety data [51].

Inclusion Criteria for all study participants: (1) Adult women (≥18 years of age) with a histologically confirmed gynecological malignancy (cervical, endometrial, vulvar, or vaginal), of any stage, who have completed a course of pelvic RT and/or CT. (2) Ability to understand and willingness to sign the study-specific Informed Consent Form. Additional criteria for inclusion in the OT Group: Presence of chronic R/CIPT, with a duration of ≥3 months following conventional symptomatic management. A toxicity grade of 2 (moderate symptoms; limiting instrumental activities of daily living) or higher, as per the CTCAE v5.0.

Exclusion Criteria for all study participants: any of the following will preclude inclusion: (1) Failure to meet all the inclusion criteria specified above. (2) Unwillingness or inability to provide written informed consent for study participation. (3) Presence of active inflammatory bowel disease (e.g., Crohn’s Disease, Ulcerative Colitis) or a history of major gastrointestinal resection (excluding appendectomy) that could significantly alter gut anatomy and microbiota. (4) Any uncontrolled intercurrent illness or psychiatric condition that, in the investigator’s opinion, would limit compliance with study requirements or interfere with the interpretation of results.

Withdrawal Criteria. Participants will be withdrawn from the study under the following circumstances: (1) Voluntary Withdrawal: At the patient’s own request, at any time and for any reason, without any penalty or impact on their future clinical care. (2) Loss to Follow-up: Inability to contact the patient or their failure to attend scheduled study visits. (3) Clinical Decision: Withdrawal by the principal investigator due to the occurrence of an adverse event, intercurrent illness, or any other clinical reason that justifies discontinuation for the patient’s well-being or the integrity of the study. (4) Protocol Violation: Significant deviation from the study protocol.

All patients, regardless of their decision to participate, decline, or withdraw from the study, will continue to receive standard oncological care and follow-up from their treating physicians. Withdrawal from the study will not affect their access to routine or compassionate medical treatments, including ozone therapy if clinically indicated.

Microbiological & Biomarker Endpoints: Primary Microbiological Endpoint: Changes in gut microbiota composition and diversity. Method: 16S rRNA gene sequencing (Illumina MiSeq platform) of stool samples. Analysis: Differential abundance of key taxa (e.g., Faecalibacterium, Roseburia, Akkermansia vs. Proteobacteria), and alpha/beta-diversity indices. Biomarker Endpoints: Serum samples will be cryopreserved at −80 °C in a registered biobank for subsequent batch analysis of inflammatory cytokines and oxidative stress markers.

Clinical Endpoints: Primary Clinical Endpoint: Pelvic and gynecological toxicity grade measured by: (1) CTCAE v5.0 scale, and (2) EORTC QLQ-CX24 module (cervical cancer-specific). Secondary Clinical Endpoints: (1) Quality of Life (QoL) measured by: EORTC QLQ-C30 (for cancer patients) and EQ-5D-5L™ (for the general population), (2) Psychological Status: Hospital Anxiety and Depression Scale (HADS), and (3) serum inflammatory/oxidative stress biomarker analysis.

Methodology: Sampling Schedule, Assays, and Statistical Approaches. Study Design: Prospective, observational study. Sample Size: 38 patients (19 with toxicity and OT, 19 matched controls without toxicity). Sampling Schedule: (1) R/CIPT Group: Baseline (pre-ozone) and post-treatment (after the final ozone session). (2) Control Group: A single time point during a routine follow-up visit.

Assays: Microbiota: Stool samples for DNA extraction and 16S rRNA sequencing. Inflammatory cytokines and oxidative stress markers: serum samples. The researchers analyzing the stool and serum samples will be blinded regarding the sample’s group assignment (non-toxicity control, pre-ozone, post-ozone).

The analysis of serum biomarkers will encompass a comprehensive panel to evaluate both oxidative stress and inflammatory status. 1. Oxidative Stress Biomarkers: The assessment will include global oxidative stress markers such as FRAP (Ferric Reducing Ability of Plasma), which measures antioxidant capacity; dROMs (Reactive Oxygen Metabolites), which determines the peroxidation potential; and BAP (Biological Antioxidant Potential). Specific molecular biomarkers will also be quantified, including the activity of the key antioxidant enzymes Superoxide Dismutase (SOD) and Catalase (CAT), the levels of reduced glutathione (GSH) as a critical cellular antioxidant, Advanced Oxidation Protein Products (AOPP) as a marker of protein damage, and ROOH (Total Hydroperoxides) to assess the primary oxidation products of lipids and other molecules. 2. Inflammatory Biomarkers: The inflammatory profile will be characterized by measuring a panel of Pro-inflammatory Cytokines: Tumor Necrosis Factor-alpha (TNF-α), Interleukin-6 (IL-6)**,** Interleukin-1 beta (IL-1β), and Interleukin-8 (IL-8/CXCL8)**.** Furthermore, systemic inflammation will be evaluated through established clinical markers: C-Reactive Protein (CRP) and VES (Erythrocyte Sedimentation Rate)**.**

Statistical Approaches: Primary Analysis: Non-parametric tests will be used due to the small sample size (Wilcoxon signed-rank for pre/post ozone, Mann–Whitney U for inter-group comparisons). Spearman correlations for microbiota-clinical parameter associations. All variables will be described as medians and quartiles 1–3 (percentiles 25–75%). *p*-values less than 0.05 will be considered significant, and all hypothesis tests will be conducted two-sided. Software: R (v4.5.0 or higher).

This study is the first to prospectively explore the novel hypothesis that the clinical benefits of rectal OT for chronic R/CIPT could be mediated, in part, through gut microbiota modulation. The proposed mechanistic pathway is: Ozone → Attenuation of local oxidative stress and inflammation (via Nrf2 activation and NF-κB inhibition) → Restoration of a healthy mucosal microenvironment → Promotion of commensal bacteria (e.g., SCFA producers) and suppression of pathobionts → Improved barrier function and reduced systemic inflammation → Clinical improvement in toxicity and Quality of Life.

This approach is fundamentally different from strategies like generic probiotics, as it does not introduce exogenous species but may instead act by “weeding and seeding” the local environment to favor the regrowth of beneficial autochthonous microbiota. If successful, OT could emerge as a novel, cost-effective modality for managing this devastating complication, potentially used alone or synergistically with targeted prebiotics or probiotics (Table 1).

This initial study has inherent limitations. (1) Its observational design and limited sample size (*n* = 38) prevent definitive causal conclusions. (2) The control group has only a single time point measurement (because a routine follow-up visit will be used). However, this sample should be enough for an initial comparison between patients with or without chronic R/CIPT. (3) The initial microbiota analysis is based on 16S rRNA sequencing. The analysis of microbial metabolites (metabolomics), particularly the measurement of SCFA and other tryptophan derivatives, would be highly interesting in the stool samples. However, due to the pilot nature of the study and the need to first establish whether significant changes exist in the community structure (diversity and differential abundance of key taxa such as Proteobacteria or butyrate producers), we have established 16S sequencing as the initial method of choice and have deferred future multi-omic studies for subsequent investigation, should they be warranted. (4) Main potential confounder factors that could significantly alter gut microbiota will be specifically recorded, such as use of systemic antibiotics within 4 weeks prior to the baseline sample collection, or regular use of probiotics (≥4 times per week) within 2 weeks prior to baseline sampling. However, because this is an “observational study”, no modifications will be made to the patient’s routine management.

To build upon these findings, future directions should include: (1) progressing to a randomized, sham-controlled trial (RCT); (2) incorporating multi-omics approaches like shotgun metagenomics and metabolomics; and (3) exploring the potential for personalized medicine by identifying microbial, inflammatory, and/or oxidative stress biomarkers of treatment response.

## 6. Conclusions

Pelvic RT and CT remain indispensable in the treatment of gynecological malignancies, yet their efficacy is counterbalanced by gastrointestinal, local and systemic toxicities that are increasingly linked to gut microbiota dysregulation [8]. The integration of microbiome science into oncology has highlighted the potential of targeting microbial communities as a strategy to mitigate treatment-induced morbidity and improve patient outcomes [52]. OT, through its well-documented redox-modulating, anti-inflammatory, and immunoregulatory actions, offers a promising complementary approach [10,29]. Preliminary evidence suggests that ozone administration—particularly rectal insufflation—may influence the intestinal microenvironment in ways that preserve microbial balance, reduce mucosal damage, and enhance resilience against cytotoxic stress [12,13,38]. The interplay between OT and gut microbiota in gynecological patients undergoing R/CIPT, however, remains an underexplored field. The development of well-designed clinical studies employing standardized ozone protocols, advanced microbiota profiling (16S rRNA sequencing, metagenomics, metabolomics), and robust toxicity endpoints is urgently needed. Such studies could clarify whether ozone can modulate dysbiosis, attenuate inflammatory cascades, and ultimately translate into improved quality of life and treatment adherence. In conclusion, OT may represent a novel and biologically plausible adjuvant in supportive care for gynecological cancer survivors. Its potential to interact with the gut microbiota opens a new line of investigation at the intersection of redox biology, microbiome science, and cancer therapy. Future research should aim not only to validate efficacy but also to define dose–response relationships, safety parameters, and long-term outcomes, thereby advancing the integration of OT into evidence-based oncological practice.

## Figures and Tables

**Table 1:** Schematic of the Rectal Ozone Therapy (OT) Clinical Study Design.

Stage	Key Elements	Study Groups & Sampling	Method and Endpoints
Population & Criteria	Objective: Adult women (≥18 years) with gynecological malignancy who have completed pelvic radiotherapy (± chemotherapy).	R/CIPT + OT Group (*n* = 19): With chronic pelvic toxicity (CTCAE ≥ Grade 2, ≥3 months duration). Control Cohort (*n* = 19): No pelvic toxicity, matched by age and tumor site.	Inclusion/Exclusion Criteria: Ensuring exclusion of confounding factors (e.g., active inflammatory bowel disease).
Intervention & Treatment	Intervention: Ozone Therapy (OT) via rectal insufflation with/without direct local application.	OT Group Only: Receives ≈ 40 sessions of OT over 4 months.	Ozone Regimen: Progressive dosing (10 µg/mL up to 30 µg/mL).
Sampling & Follow-Up	Design: Prospective, observational study. Blinding: Sample analysis will be blinded to group assignment.	OT Group: Stool/serum samples at Baseline (pre-ozone) and post-treatment (week 16). Control Group: A single sample taken at routine follow-up.	Confounders: Strict recording of recent use of antibiotics and probiotics.
Clinical & Biomarker Endpoints	Objective: Evaluate the effect of Ozone on toxicity and systemic status.	Clinical Assessments: Toxicity: CTCAE v5.0 and EORTC QLQ-CX24. Quality of Life (QoL): EORTC QLQ-C30 and EQ-5D-5L™. Psychological Status: HADS.	Biological Samples: Microbiota analysis (16S rRNA gene sequencing), Inflammatory Cytokines and Oxidative Stress Markers (serum).
Analysis & Conclusion	Core Hypothesis: Ozone modulates gut microbiota (weeding and seeding) → reduces inflammation → clinical improvement.	Analysis: Non-parametric tests (Wilcoxon, Mann–Whitney U) due to small sample size (*n* = 38). Significance: *p* < 0.05.	Limitation: Observational design and sample size prevent definitive causal conclusions.

Legend: R/CIPT: Radiotherapy/Chemotherapy-Induced Pelvic Toxicity; OT: Ozone Therapy. CTCAE: Common Terminology Criteria for Adverse Events (v5.0), a standardized scale for toxicity grading; EORTC QLQ-CX24: European Organization for Research and Treatment of Cancer Quality of Life Questionnaire—Cervical Cancer Module; EORTC QLQ-C30: European Organization for Research and Treatment of Cancer Quality of Life Questionnaire—Core Module (for cancer patients); EQ-5D-5L™: EuroQol-5 Dimensions-5 Levels, a standardized instrument for measuring general health-related Quality of Life; HADS: Hospital Anxiety and Depression Scale; 16S rRNA: 16S ribosomal Ribonucleic Acid gene sequencing, used to identify and quantify microbial populations; QoL: Quality of Life.

## Data Availability

Data supporting reported results in this review can be found in the respective references.

## Abbreviations

The following abbreviations are used in this manuscript:

5-FU	5-Fluorouracil
AOPP	Advanced Oxidation Protein Products
BAP	Biological Antioxidant Potential
CAT	Catalase
CRP	C-Reactive Protein
CTCAE	Common Terminology Criteria for Adverse Events
dROMs	Reactive Oxygen Metabolites
EQ-5D-5L™	EuroQol-5 Dimension-5 Level questionnaire
EORTC QLQ-CX24	European Organization for Research and Treatment of Cancer Quality of Life Questionnaire Cervical Cancer Module 24
EORTC QLQ-C30	European Organization for Research and Treatment of Cancer Quality of Life Questionnaire Core 30
FRAP	Ferric Reducing Ability of Plasma
FMT	Fecal Microbiota Transplantation
GALT	Gut-Associated Lymphoid Tissue
GI	Gastrointestinal
GSH	Reduced Glutathione
HADS	Hospital Anxiety and Depression Scale
LOPs	Lipid Oxidation Products
IL-1β	Interleukin-1 beta
IL-6	Interleukin-6
IL-8/CXCL8	Interleukin-8 / C-X-C Motif Chemokine Ligand 8
NF-κB	Nuclear Factor kappa B
Nrf2	Nuclear Factor Erythroid 2-Related Factor 2
OT	Ozone therapy
QT	Chemotherapy
QoL	Quality of Life
RCT	Randomized Controlled Trial
ROOH	Total Hydroperoxides
RT	Radiotherapy
R/CIPT	Radiotherapy/Chemotherapy-Induced Pelvic Toxicity
ROS	Reactive Oxygen Species
SCFA	Short-Chain Fatty Acid
SOD	Superoxide Dismutase
TMA	Trimethylamine
TMAO	Trimethylamine N-oxide
TNF-α	Tumor Necrosis Factor-alpha
VES	Erythrocyte Sedimentation Rate
